# Severe congenital neutropenia due to G6PC3 deficiency: early and delayed phenotype of a patient

**DOI:** 10.1186/s13223-023-00804-4

**Published:** 2023-06-09

**Authors:** Negar Moradian, Samaneh Zoghi, Elham Rayzan, Simin Seyedpour, Raul Jimenez Heredia, Kaan Boztug, Nima Rezaei

**Affiliations:** 1 School of Medicine, Tehran University of Medical Sciences, Tehran, Iran; 2 Research Center for Immunodeficiencies (RCID), Children’s Medical Center, Tehran University of Medical Sciences, Tehran, Iran; 3 Ludwig Boltzmann Institute for Rare and Undiagnosed Diseases, Vienna, Austria; 4 St. Anna Children’s Cancer Research Institute (CCRI), Vienna, Austria; 5 CeMM Research Center for Molecular Medicine of the Austrian Academy of Sciences, Vienna, Austria; 6 International Hematology/Oncology of Pediatrics Experts (IHOPE), Universal Scientific Education and Research Network (USERN), Tehran, Iran; 7 Department of Pediatrics and Adolescent Medicine, Medical University of Vienna, Vienna, Austria; 8 St Anna Children’s Hospital, Department of Pediatrics and Adolescent Medicine, Medical University of Vienna, Vienna, Austria; 9 Department of Immunology, School of Medicine, Tehran University of Medical Sciences, Tehran, Iran; 10 Network of Immunity in Infection, Malignancy and Autoimmunity (NIIMA), Universal Scientific Education and Research Network (USERN), Tehran, Iran

**Keywords:** Severe congenital neutropenia, G6PC3 deficiency, Whole exome sequencing

## Abstract

**Background:**

Severe Congenital Neutropenia type 4 (SCN4), is a rare autosomal recessive condition, due to mutations in the *G6PC3* gene. The phenotype comprises neutropenia of variable severity and accompanying anomalies.

**Case presentation:**

We report a male patient with confirmed G6PC3 deficiency presented with recurrent bacterial infections and multi-systemic complications. Our case was the first with a novel homozygous frameshift mutation in *G6PC3*. The patient demonstrated large platelets on his peripheral blood smear which is a rare presentation of this disease.

**Conclusion:**

As SCN4 patients could be easily missed, it is recommended to consider *G6PC3* mutation for any case of congenital, unexplained neutropenia.

## Introduction

Severe congenital neutropenia (SCN) is an inborn error of immunity (IEI), which is characterized by an increase susceptibility to early childhood bacterial infections. To date, more than 7 genes have been identified to be responsible for this phenotype [1, 2]. SCN subgroups and the genetic causes are summarized in Table 1.

**Table 1:** Severe congenital neutropenia subgroups and form of inheritance

Mutated gene	Inheritance	Year of discovery
*ElANE*	Autosomal dominant	1999
*GF11*	Autosomal dominant	2003
*TCIRG1*	Autosomal dominant	2014
*HAX1*	Autosomal recessive	2007
*JAGN1*	Autosomal recessive	2014
*G6PC3*	Autosomal recessive	2009
*CSF3R*	Various Autosomal recessive	1995

In 2009, Botzug et al. identified biallelic mutation in *G6PC3* gene, encoding the catalytic subunit 3 of glucose-6-phosphatase which led to SCN via dysregulation of molecular pathways resulting in the granulopoiesis arrest at the promyelocyte stage [2, 3].

Glucose-6 phosphatase catalytic subunit 3 (*G6PC3*) mutations are classified as SCN type 4 (SCN4), which is presented with multiple organ involvements such as congenital heart defects, urogenital abnormalities, superficial vein visibility, in addition to severe neutropenia (absolute neutrophil count less than 0.5 × 10^9^/L) [4, 5].

Most patients suffering from SCN type 4, respond to a colony-stimulating recombinant human granulocyte colony stimulating factor (rhGCSF) treatment, which raises neutrophil counts and decreases infection frequency and severity [6]. However, patients may remain at risk for both infectious complications and clonal hematopoiesis conditions due to the treatment [7]. G6PC3-deficient patients are not considered to be at risk for malignant transformation. Hematopoietic stem cell transplantation (HSCT) might be considered for these patients only in cases of unusual severity and/or insufficient response to G-CSF.

Here we report a male patient with confirmed G6PC3 deficiency presented with recurrent bacterial infections and multi-systemic complications. Following our case presentation; we will briefly review SCN type 4 different aspects, the disease’s classification, clinical manifestation, prevalence, and its current management.

## Case presentation

**Fig. 1:**
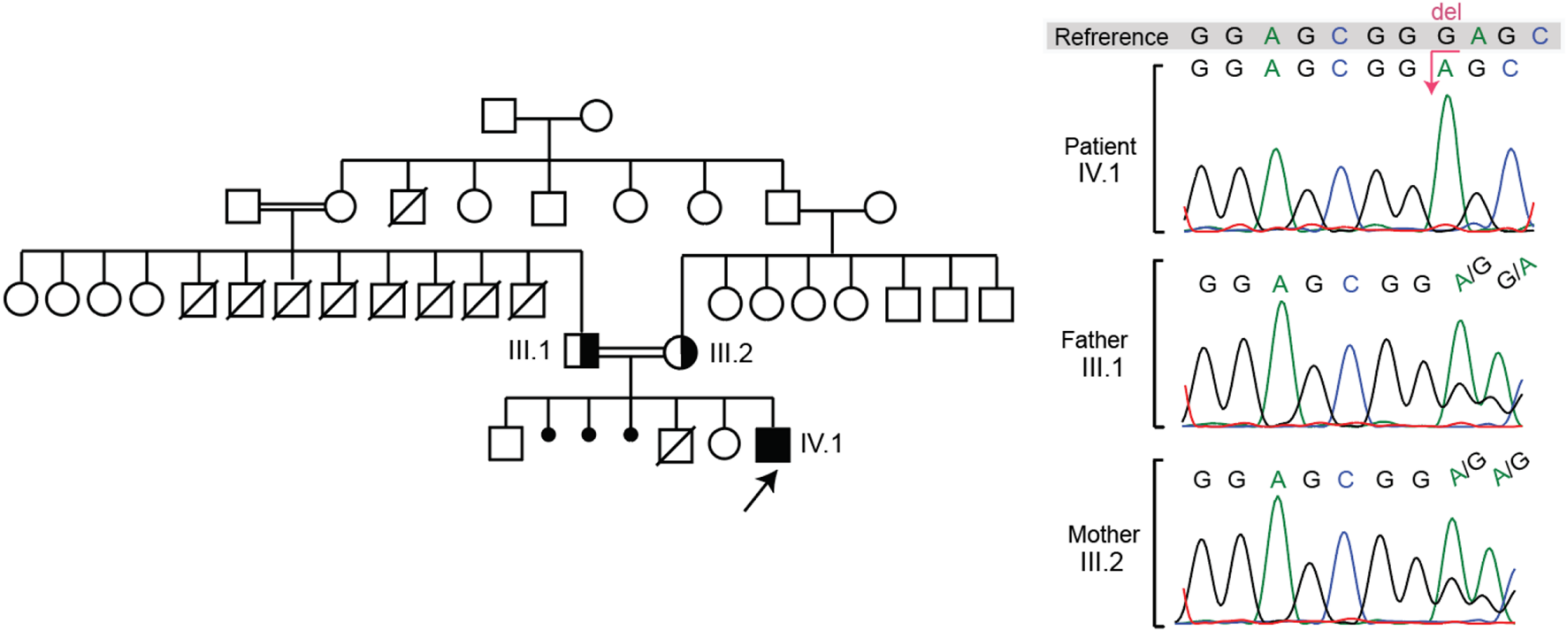
Pedigree of the patient (IV.1) and chromatograms of sanger validation of the G6PC3 variant in the patient and his parents (III.1 and III.2)

The patient is a male who was born term and vaginally with 2500 g of weight and with no pregnancy complications. He was born to an Iranian couple who are first-degree cousins. The patient has two healthy siblings (a sister and a brother) and there has been a history of a 10 day infant loss in addition to three abortions in the family, without any identified cause. Additionally, there was a history of eight infants (or early child) deaths in the paternal family (father’s siblings); all of which were males, and the patient’s father is the only survivor. The patient’s pedigree is illustrated in Fig. 1.

The patient presented with facial, rectal, and thoracic abscesses at birth and had a history of skin abscess, fever, recurrent otitis leading to hearing loss. His cardiovascular system reports revealed ventricular septal defect (VSD) at birth, which was restricted spontaneously. Additionally at birth the patient had inguinal herniation on both sides, which had been repaired by surgery. He had several pneumonia episodes which made him bed ridden, in addition to chronic diarrhea and severe gingivitis in his early childhood. No history of bronchitis, meningitis, endocrine complications or osteomyelitis as well as organ abscess were reported for the patient. However, he suffered from recurrent fever and diarrhea together with multiple fatigue complains to date. The present and past signs and symptoms of the case is summarized in Table 2.

**Table 2:** Present and past signs and symptoms of the patient

**Fig. 2:**
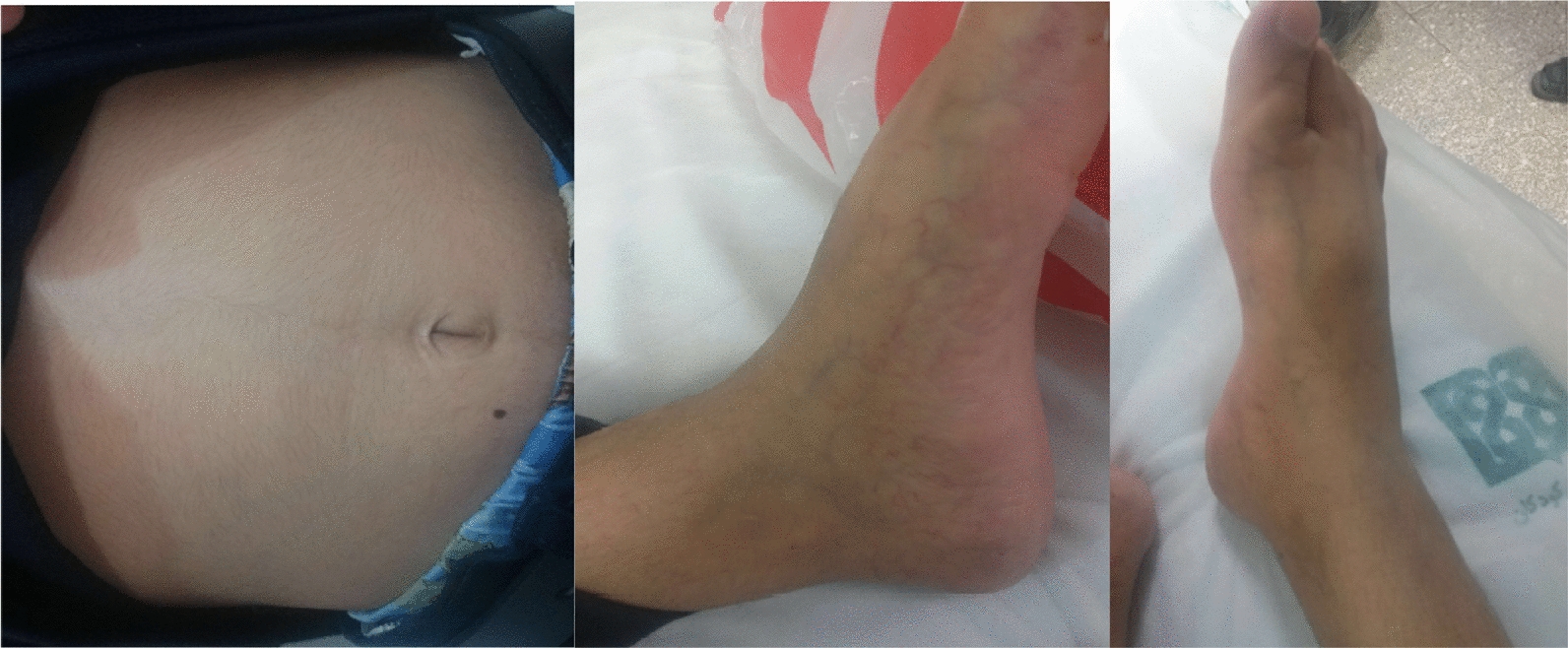
Telangiectasia in abdomen and extremities of lower limbs

**Fig. 3:**
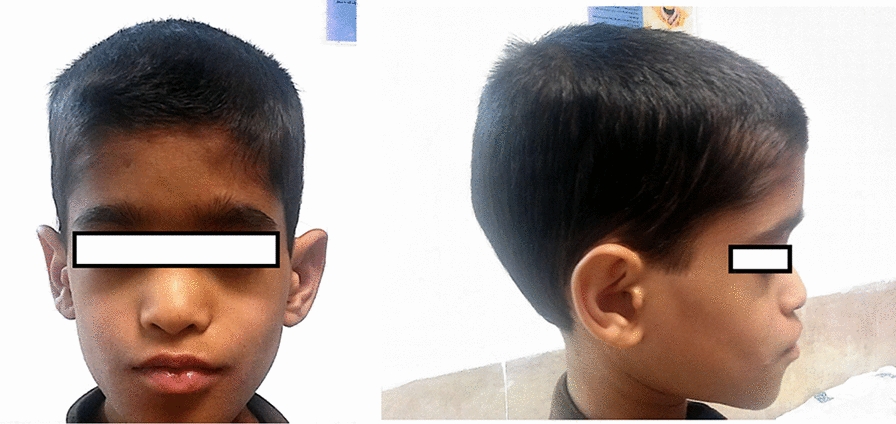
The patient’s facial features. Low set ears and low hair-line

**Cardiovascular**
• Ventricular septal defect at birth which was restricted spontaneously
• Easily fatigued
• Abdomen and lower limbs telangectasia (Fig. 2)
**Urogenital**
• Inguinal herniation on both sides at birth which had been repaired uncomplicated
**Skin**
• Facial, rectal and thoracic abcess at birth
• Visible superficial veins
**ENT**
• Recurrent otitis leading to current hearing loss
**Facial features**
• Low-set ears (Fig. 3)
• Low hairline (Fig. 3)
• Broad nasal bridge
**Respiratory system**
• History of several episodes of pneumonia that made him bed-ridden but none were severe
• No history of bronchitis
**Gastrointestinal**
• Current chronic diarrhea
**Other**
• Recurrent fever
• Failure to thrive

The patient was referred to our center at the age of nine due to his severe neutropenia, while he was previously diagnosed as an SCN case since the age of four and G-CSF treatment was started for him by the age of six. Prior to G-CSF prescription, he suffered from several bacterial infections, most of which were resolved thereafter. The patient’s bone marrow analysis before the start of G-CSF therapy, was reported normo-cellular and M/E ratio was normal, all myeloid series were seen, however myeloid lineage maturation arrest was confirmed. All erythroid series were seen according to his report without any erythropoiesis. The bone marrow analysis also reported normal lymphoid megakaryocytes.

At his referral (9 years of age), the patient was receiving a complete dose of pegylated G-CSF (containing 6 mg/0.6 ml) every 11 days considering that his weight was > 45 kg. On his physical exam at the age of nine, the patient had clear slow growth and failure to thrive (height below the 5th percentile). His facial features revealed low set ears, low hairline, and a broad nasal bridge. He has also presented with vivid prominent superficial vascular system on his stomach and visible superficial veins on his lower limb. Anal fissures were observed in the patient reminiscent of previous abscesses. Routine investigations revealed severe neutropenia for the patient (4% neutrophils, absolute neutrophil count 460/µL). His Complete Blood Count (CBC) revealed anemia with anisocytosis, microcytosis, hypochromia, lymphopenia, and thrombocytopenia. The CBC and serum immunoglobulin test results are summarized in Table 3. The result of Immunophenotyping from the same time is shown in Table 4.

**Table 3:** Complete Blood Count and serum immunoglobulin levels of the patient

	3 days after G-CSF treatment		2 weeks after G-CSF treatment		Age-related Ref. values
	Absolute number	Percentage	Absolute number	Percentage	
WBC	1.94 (10^3^/uL)		9.25 (10^3^/uL)		4.0–10.3 (10^3^/uL)
RBC	3.80 (10^6^/uL)		4.96 (10^6^/uL)		4.4–5.6 (10^6^/uL)
HGB	10.2 (g/dL)		11.9 (g/dL)		12.3–16.3 (g/dL)
HCT	31.0 (%)		34.8 (%)		35.4–43.4 (%)
MCV	81.6 (fL)		70.2 (fL)		76.9–87.2 (fL)
MCH	26.8 (pg)		24 (pg)		25.8–30.0 (pg)
MCHC	32.9 (g/dL)		34.2 (g/dL)		32.3–35.9 (g/dL)
PLT	54 (10^3^/uL)		193 (10^3^/uL)		216–469 (10^3^/uL)
RDW-SD	56.6 (fL)		33.5 (fL)		
RDW-CV	19.7 (%)		13.4 (%)		11.9–14.3 (%)
NEUT	1.03 (10^3^/uL)	53.1 (%)	1.52 (10^3^/uL)	16.5 (%)	32.3–64.1 (%)
LYMPH	0.62 (10^3^/uL)	32.0 (%)	6.42 (10^3^/uL)	69.5 (%)	26.1–57.5 (%)
MONO	0.28 (10^3^/uL)	14.4 (%)	1.14 (10^3^/uL)	12.3 (%)	3.9–10 (%)
EO	0.00 (10^3^/uL)	0.0 (%)	0.17 (10^3^/uL)	1.8 (%)	0.4–7.5 (%)
Baso	0.01 (10^3^/uL)	0.5 (%)	0.0 (10^3^/uL)	0.0 (%)	0.1–1.0 (%)
IgM	156 (37–286)				
IgG	2881 (639–1349)				
IgA	140 (42–295)

**Table 4:** Immunophenotyping results (July 26, 2017)

Lymphocyte marker	Lymph%	Lymph count	Normal values% (7–12 years old)
CD3	64	742	60–76
CD4	32	371.2	31–47
CD8	32	371.2	18–35
CD19	10	116	13–27
CD16	22	255.2	4–17
CD56	56	650	4–17

No neutrophil function test was done for the patient due to unavailability of such assays at the local diagnostic lab. During the patient’s follow-up visits, giant platelets were reported in the latest blood smear, which is a rare presentation for these patients.

The patient was suspected to SCN type 4, due to his phenotypic features and lab data. To identify the underlying genetic defect in the patient, we sequenced a panel of Primary Immunodeficiency Diseases (PID) genes by means of next generation sequencing (NGS). The analysis of exome data revealed a novel homozygous frameshift mutation in *G6PC3* in the position 194 of the protein (c.583del, p.Glu195SerfsTer2), with a high CADD (Combined Annotation Dependent Depletion) score of 32, which shows that the mutation is probably pathogenic. The panel sequencing detail is displayed in Table 5. Both parents were heterozygous for the mutation and the chromatograms are displayed in Fig. 1.

**Table 5:** Panel sequencing results

Gene name	Chromosome	Position	ID	Reference	Alternative	Mutation type	AA change	Transcript_ID	frequency in gnomAD	Depth	SIFTcat	PolyPhenCat	CADD
Homozygous variants from PID and HM Panel													
* G6PC3*	17	42152722		CG	C	Frame shift	R194	ENST00000269097		163	NA	NA	32
Heterozygous variants from PID and HM panel													
* PIEZO1*	16	88782507		C	T	Missense	G2384S	ENST00000301015	0.00003269 (5 het)	203	Tolerated	Benign	13.9
* HK1*	10	71075748	rs374788115	G	A	Missense	G13D	ENST00000298649	0.00002852 (8 het)	115	NA	NA	11.54
* FCGR3A*	1	161518448		C	G	Missense	V64L	ENST00000367969	NA	83	Tolerated	Probably_damaging	22.5
* EDAR*	2	109529140		G	A	Missense	H175Y	ENST00000376651	NA	84	Tolerated	Benign	16.24
* PIEZO1*	16	88804732		C	T	Missense	A251T	ENST00000301015	0.00002828 (4 het)	183	Tolerated	Benign	10.37

## Discussion

We herein report a case of G6PC3 deficiency, presenting with recurrent bacterial infections and multi-systemic complications, bearing a novel homozygous frameshift mutation in *G6PC3.* Our presented case was worth reporting due to the novelty of the mutation. Furthermore, our patient has developed almost all phenotypes of G6PC3 deficiency which is rare and they fit well with the known phenotypic data of the disease, which is reviewed in the following lines.

G6PC3 deficiency is characterized by extreme congenital neutropenia that occurs in a phenotypic spectrum from (a) Isolated severe congenital neutropenia (non-syndromic) to (b) Classic G6PC3 deficiency (syndromic), which is severe congenital neutropenia plus cardiovascular and/or urogenital abnormalities [8].

The most prominent hematologic feature of G6PC3 deficient patients is persistent severe neutropenia (neutrophils count below 0.5 × 10^9^/L) which is the main phenotype of the disease. Apart from neutropenia, intermittent thrombocytopenia is often observed in nearly two-thirds of the patients. Furthermore, lymphopenia and scarce giant platelets are among the other features detectable in the CBC of severe cases [3, 9–12]. Bone marrow examination of such patients may reveal maturation arrest in the myeloid lineage; however, some of the G6PC3 deficient patients have hyper- or normo-cellular bone marrow [13].

According to the literature, 77% of G6PC3 deficient cases, present congenital cardiovascular defects with atrial septal defects being the most common anomaly. Some patients can benefit from correcting surgery while others benefit from conservative treatment [7, 14].

Another prevalent sign which is common in affected children between late infancy and early childhood is prominent superficial venous pattern [15]. This pattern can be seen on the trunk, extremities and sometimes on the head. In our patient visible superficial veins was detectable on his abdomen and extremities of the lower limbs as depicted in Fig. 2 [7, 15].

Urogenital defects are another frequent symptom that has been reported in 43.8% of G6PC3 deficient patients. This anomaly is significantly more common in affected males with cryptorchidism being the most common presentation [7, 14]. Other urogenital malformations reported in these patients include severe vesicoureteral reflux, hydronephrosis, poor renal cortico-medullary differentiation, hypospadiasis and small kidneys. Some of the affected cases can benefit from correcting surgery.

Gastrointestinal tract is another affected organ with an unknown etiology. Pre-clinical research has demonstrated that increased anti-granulocyte colony stimulating factor (G-CSF) antibodies are associated with ileitis. Also lack of granulocyte macrophage colony stimulating factor (GM-CSF), shown to alter epithelial associations with intestinal microbes contributing to the inflammation of the intestine [16, 17].

The disease shown to affect central nervous system as well. Mild learning difficulties and bilateral brain atrophy in MRI, have been reported in various studies [3, 7].

Intrauterine growth restriction (IUGR), failure to thrive (FTT), and poor postnatal growth are commons features of G6PC3 deficiency, which could be either secondary to repeated infections or a primary phenotype [8].

Additionally, variable facial dysmorphologic features, including frontal bossing, thick lips, broad nasal bridge, and prognathism are reported for these patients [3, 7, 18]. The facial dysmorphism observed in the  presented patient is depicted in Fig. 3. Other malformations include inguinal and umbilical hernias, cabal gated toes and redundant skin folds of the neck.

An approximate marginal incidence of congenital neutropenia is 6 per million [19]. Based on data from 650 patients with severe congenital neutropenia registered in the European and North American Branches of the Severe Chronic Neutropenia International Registry, G6PC3 deficiency accounts for 2% of SCN. Yet, the frequency of SCN type 4 varies greatly from one population to another [14, 20]. For instance, G6PC3 deficiency is the most common cause of severe congenital neutropenia in Israel; 25% of diagnosed SCN patients [21].

G-CSF is largely prescribed to control the neutropenia in G6PC3 deficient patients. G-CSF treatment showed to increase the number of neutrophils, prevent infections and improve patients’ quality of life [6, 14]. However, it is not always effective, as this therapeutic approach failed to control infections in some patients even at high doses [20]. It has been reported recently in a study that G-CSF, in vivo could not enhance neutrophil function [22].

In comparison, moderately infected patients may not necessarily require G-CSF medication and may merely be treated with prophylactic antibiotics [14].

It has also recently been reported that empagliflozin, an inhibitor of the renal glucose cotransporter sodium glucose cotransporter 2 (SGLT2) which is an anti-diabetic drug, could be considered as an alternative therapy for neutropenia cases. SGLT2 inhibitors have been shown to improve neutrophil function in patients affected with 1, 5-anhydroglucitol-6-phosphate (1,5AG6P). Clinically, symptoms of mucosal lesions, frequent infections and inflammatory bowel disease resolved, and no patients had symptoms of hypoglycemia [23]. This treatment has been also very recently demonstrated to improve and normalize neutrophil counts in two G6PC3 deficient children [24].

Studies of G6PC3-deficient neutrophils revealed dysfunction of these cells in patients. It has been shown that these neutrophils are prone to apoptosis, and they express higher levels of activation markers (CD11b, CD66b, and CD14) [25]. Although HSCT is believed to be the effective approach to restore the function of neutrophils, G-CSF shown partial improvement of neutrophil function in some [6, 26], but not in all studies [22].

G6PC3 deficiency syndrome was first described by genotyping and association study of 2 consanguineous Armenian families with a total of five SCN affected children and accompanying systemic heart defects, urogenital anomalies, and venous angiectasia on extremities and trunk symptoms [4]. Analysis of these families revealed a homozygous missense mutation in exon 6 of the gene, on chromosome 17q21, encoding G6PC3 [4]. Our patient genomic analysis revealed a novel homozygous frame-shift mutation due to a single G nucleotide deletion in exon 5 of the *G6PC3* gene (Table 4). A missense G to A mutation in the same position exists in gnomAD (genome aggregation database) and it is linked to SCN type 4 in Clinvar but we could not find the same frameshift mutation in the literature. The detected deletion causes premature termination, resulting in a truncated, probably non-functional protein, which leads to a variety of symptoms.

Intermittent thrombocytopenia is a frequent feature of *G6PC3* mutation. Our patient had severe intermittent thrombocytopenia in addition to giant platelets and a high mean platelet volume (MPV) in his peripheral blood smear. To our knowledge, to date, only three other cases have been reported [3, 27] with giant platelets. We should mention here that it is not completely clear whether thrombocytopenia and giant platelets are due to the gene defect or a secondary phenomenon of the ongoing recurrent infections. Our patient also presented with other symptoms including lymphopenia, neutropenia, anemia, recurrent pneumonitis, cardiac features, visible superficial veins, chronic diarrhea, recurrent fever, facial features, urogenital features, and developmental delay. These symptoms could be an explanation for delayed SCN diagnosis in our patient during the first years of his life.

Various clinical features that mentioned here could be considered for early diagnosis of the patient and positively affect therapeutic consequences. The presented G6PC3 deficient case had normo-cellular bone marrow post G-CSF treatment, he had an increase in neutrophil count, improved growth, and a complete resolution of rectal and chest abscesses after the treatment.

To sum up, cases similar to our patient, are ideal disease model not only from the clinical point of view and for educational purposes in clinical practice, but also from the research point of view, as they are perfect examples of nature to study the gene function. In the presented case further investigation is required to elucidate whether the detected thrombocytopenia and was the result of *G6PC3* mutation, or it is secondary to other complications of the disease.

## Data Availability

Not applicable.

## Abbreviations

SCN4: Severe Congenital Neutropenia type 4
SCN: Severe congenital neutropenia
G6PC3: Glucose-6 phosphatase catalytic subunit 3
rhGCSF: Colony-stimulating recombinant human granulocyte factor
VSD: Ventricular septal defect
CBC: Complete blood count
PID: Primary immunodeficiency diseases
CADD: Combined annotation dependent depletion
G-CSF: Granulocyte colony-stimulating-factor
IUGR: Intrauterine growth restriction
FTT: Failure to thrive
SGLT2: Sodium glucose cotransporter 2
